# Genome-wide association study reveals genomic regions controlling root and shoot traits at late growth stages in wheat

**DOI:** 10.1093/aob/mcz041

**Published:** 2019-04-09

**Authors:** Long Li, Zhi Peng, Xinguo Mao, Jingyi Wang, Xiaoping Chang, Matthew Reynolds, Ruilian Jing

**Affiliations:** 1 National Key Facility for Crop Gene Resources and Genetic Improvement/Institute of Crop Science, Chinese Academy of Agricultural Sciences, Beijing, China; 2 International Maize and Wheat Improvement Center, Texcoco, Mexico

**Keywords:** Root, shoot, genome-wide association study, Wheat 660K SNP Array, genetic relationship, Triticum aestivum

## Abstract

**Background and Aims:**

Root system morphology is important for sustainable agriculture, but the genetic basis of root traits and their relationship to shoot traits remain to be elucidated. The aim of the present study was to dissect the genetic basis of root traits at late growth stages and its implications on shoot traits in wheat.

**Methods:**

Among 323 wheat accessions, we investigated phenotypic differences in root traits at booting and mid-grain fill stages in PVC tubes, shoot traits including plant height (PH), canopy temperature (CT) and grain yield per plant (YPP) in a field experiment, and performed a genome-wide association study with a Wheat 660K SNP Array.

**Key Results:**

Deep-rooted accessions had lower CT and higher YPP than those with shallow roots, but no significant relationship was identified between root dry weight and shoot traits. Ninety-three significantly associated loci (SALs) were detected by the mixed linear model, among which three were hub SALs (*Co-6A*, *Co-6B* and *Co-6D*) associated with root depth at both booting and mid-grain fill stages, as well as CT and YPP. Minirhizotron system scanning results suggested that the causal genes in the three SALs may regulate root elongation in the field. The heritable independence between root depth and PH was demonstrated by linkage disequilibrium analysis. The YPP was significantly higher in genotypes which combined favourable marker alleles (FMAs) for root depth and PH, suggesting that a deep root and shorter plant height are suitable traits for pyramiding target alleles by molecular marker-assisted breeding.

**Conclusions:**

These results uncovered promising genomic regions for functional gene discovery of root traits in the late growth period, enhanced understanding of correlation between root and shoot traits, and will facilitate intensive study on root morphology and breeding through molecular design.

## INTRODUCTION

Wheat (*Triticum aestivum* L.) is a worldwide staple food grain. As a result of global climate change and scarcity of water and nutrients, abiotic stresses increasingly curtail wheat yield (Barnabas *et al.*, 2008; Mondal *et al.*, 2015*b*). To meet predicted global demand (gains in yield of 50 % in approx. 20 years), breeding wheat varieties with eurytopicity and efficient use of water and nutrients are urgently required (Tester and Langridge, 2010; Odegard and van der Voet, 2014). Roots are primary organs that absorb water and minerals, and perceive stress signals from the soil; their characteristics determine absorption capacity and response to stress (Lynch, 2007; Y. Fang *et al.*, 2017). Therefore, modifying the distribution of roots in the soil to optimize water and nutrient uptake was expected to be a major breakthrough for the second Green Revolution partly based on tolerance of low soil fertility (Ehdaie *et al.*, 2012).

Despite the importance of root traits in crop performance, they have rarely been used as selection criteria in breeding. One of the main reasons is that the relationships among root traits and other agronomic traits, which is the prerequisite for root character optimization and ideotype design, remains to be elucidated (Wasson *et al.*, 2014). Modelling indicated that proliferation at depth of wheat roots could in theory improve yield in environments where water is available deeper in the soil profile at grain fill stage (Manschadi *et al.*, 2006; Lilley and Kirkegaard, 2011). Kirkegaard *et al.* (2007) demonstrated that a 30 cm increase in root depth into the sub-soil could capture an extra 10 mm of deep soil water. Genetic differences in eight wheat sister lines under drought stress confirmed a relationship between deep roots and yield (Lopes and Reynolds, 2010). However, Ma *et al.* (2008) found that the water use efficiency (WUE) of winter wheat was improved by reducing the root biomass in the upper soil layer. Previous studies of relationships between root and shoot traits have generally been performed using a small number of cultivars often at relatively early growth stages, therefore ignoring phenotypic variation during essential growth stages for yield such as booting and flowering (Watt *et al.*, 2013). Therefore, studies using sizable genetic populations focused at later growth stages should provide information of more practical value for breeding efforts.

Another main reason hindering the utilization of root traits in breeding is that roots are hidden in soil and obscured from direct observation. The rapid development of biological techniques, such as marker-assisted selection, provides an opportunity to overcome this obstacle. For instance, a molecular marker for a major quantitative trait locus (QTL; *qWT_Gm03*) associated with the number of adventitious roots in soybean was validated in near-isogenic backgrounds as a reliable marker for waterlogging-tolerant breeding in multiple environments (Ye *et al.*, 2018). In rice, overexpression of *DRO1* shows a benefit for drought tolerance by increasing deep rooting (Uga *et al.*, 2013). Overexpression of *PSTOL1* significantly increased total root surface, enabling plants to acquire more phosphorus and gain yield in phosphorus-deficient soil (Gamuyao *et al.*, 2012). Pinto *et al.* (2010) identified QTLs associated with canopy temperature in wheat and these were later shown to be associated with the responsiveness of root growth under both heat and drought stress (Pinto and Reynolds, 2015). Other QTLs/genes associated with root traits are comprehensively reviewed by Meister *et al.* (2014) and Hochholdinger *et al.* (2018). However, the genetic dissection of root traits in wheat is relatively limited, mainly because the wheat genome is much larger and more complicated than that of other crops. Improvement of the accuracy and integrity of whole-genome sequencing facilitates the development of single nucleotide polymorphism (SNP) arrays in wheat, which is a specific and high-throughput tool for genotyping (Cavanagh *et al.*, 2013; Wang *et al.*, 2014). The newly developed Wheat 660K SNP Array can detect >630 000 SNPs with advantages of genome specificity and high efficiency (Zhou *et al.*, 2018). Then, by performing genome-wide association studies (GWAS), major QTLs/alleles related to root traits in wheat are expected to be identified, which is the motivation for functional gene discovery and genetic network construction. The dissection of a specific trait is insufficient for molecular breeding, because many complex traits tend to be tightly integrated and change together, resulting in heritable covariation (C. Fang *et al.*, 2017). The genetic network can help us to select independent genes with pyramiding potential, and pleiotropic genes with major effect (Guo *et al*., 2017, 2018). One noteworthy example is *VRN1*, a key regulator of ﬂowering behaviour in cereals (Deng *et al.*, 2015), which was recently reported to modulate overall plant morphology in wheat, thereby regulating the balance between shoot and root architecture (Voss-Fels *et al.*, 2018). Therefore, more investigations to uncover the genetic relationship among root and shoot traits are urgently required.

Here, we investigated phenotypic differences in root and shoot traits of 323 wheat accessions at late growth stages, and performed GWAS using the Wheat 660K SNP Array. The aims of the study were to: (1) investigate the phenotypic association between root and shoot traits at late growth stages; (2) dissect the genetic basis of root traits at late growth stages and its implication on shoot traits; and (3) identify hub SALs (significantly associated loci) controlling root and shoot traits.

## MATERIALS AND METHODS

### Plant materials

The germplasm used in this study consisted of 323 winter wheat accessions (Supplementary Data Table S1), most of whose flowering dates occurred within 1 week (Supplementary Data Fig. S1). The accessions include 12 landraces, 36 advanced lines and 275 modern varieties, mainly planted in the Yellow and Huai River Valleys Facultative Wheat Zone and the Northern Winter Wheat Zone in China.

### Root trait phenotyping

**Fig. 1.**
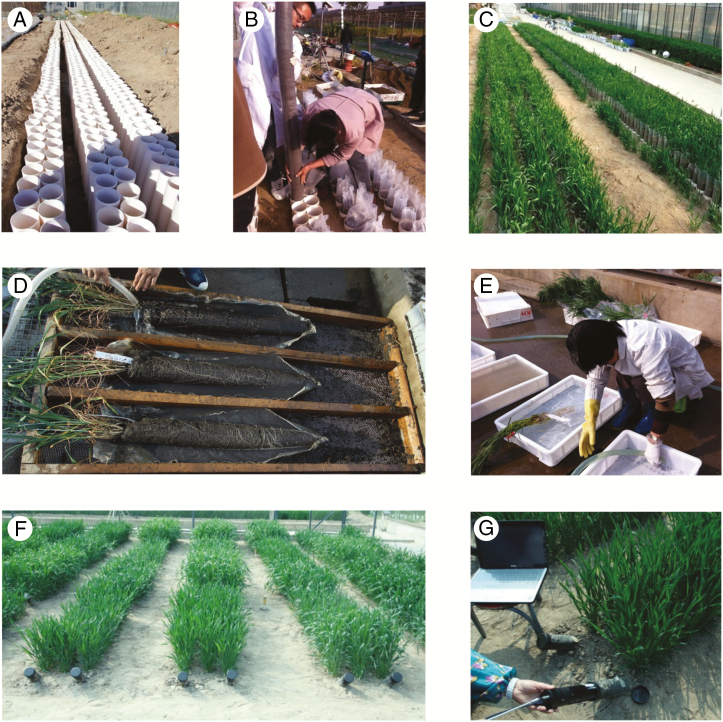
Root trait phenotyping. (A) PVC pipes were buried in a soil pit. (B) Soil-filled bags were inserted into each tube. (C) Plants at the jointing stage. (D) Roots were preliminarily washed with a sieve. (E) Roots were further washed in plastic boxes. (F) Minirhizotron pipes were buried in soil with a 45° angle of inclination to measure root traits in each trial plot. (G) Minirhizotron images were collected by a CI-600 Roots Monitoring System.

**Fig. 2.**
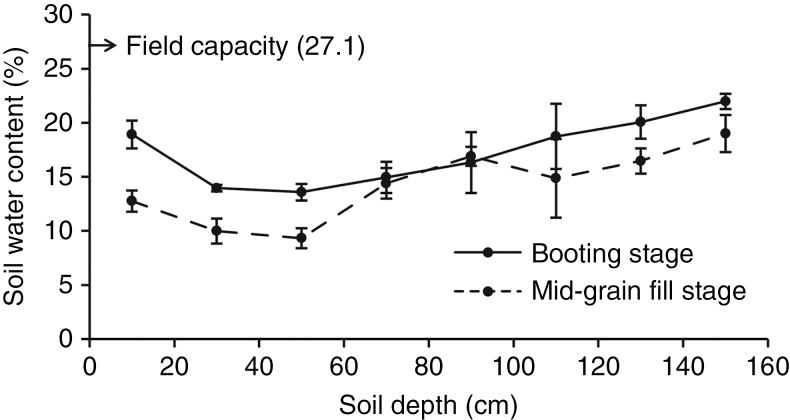
Average soil water contents in 0–160 cm soil layers at the booting and mid-grain filling stage. The arrow indicates average field capacity, and error bars denote the s.d.

All accessions were grown in polyvinyl chloride (PVC) pipes at the Institute of Crop Science Experimental Station in Beijing (116°28′E, 39°48′N) from the beginning of October 2014 to May 2015, in a randomized complete block design with three replicates. The root traits were measured at the booting and mid-grain fill stages. The phenotyping workflow was as follows: (1) PVC pipes (11 cm in diameter and 3.2 mm thick, 1.0 m long for plants sampled at booting, 1.8 m long for plants sampled at mid-grain fill) were buried in a soil pit (Fig. 1A); (2) polyethylene (PE) bags matching the PVC pipe lengths (10 cm in diameter and 0.2 mm thick) were filled with soil with bulk density 1.13 g cm^–3^, placed in the PVC pipes and watered to 80 ± 5 % of field capacity (Fig. 1B). The soil was a fluvo-aquic loam with basic fertility characteristics of organic matter 19.1 g kg^–1^, total N 1.04 g kg^–1^, available phosphorus 18.5 mg kg^–1^ and available potassium 131.24 mg kg^–1^; (3) eight seeds were sown in each pipe; (4) seedlings were thinned to three plants; (5) each PE bag was weighted before winter, at the jointing, booting and flowering stages, to evaluate moisture loss, and re-watered to 80 ± 5 % field capacity; (6) PE bags were withdrawn from the PVC pipes at the booting and mid-grain fill stage (Fig. 1C); (7) root depths were recorded according to the deepest root tip; (8) the bags were split, and soil columns were carefully washed to obtain the complete root system in a sieve of 0.5 × 0.5 cm (Fig. 1D); and (9) roots were washed again in plastic boxes (Fig. 1E), sheared from the basal stem and dried for measurement of dry weight.

To measure root traits of accessions in the field, 60 minirhizotron pipes (2 m in length, 64 mm in diameter) were buried in soil with a 45° angle of inclination. There were two pipes per plot, set 20 cm apart. The space between plots was 60 cm (Fig. 1F). Ten accessions selected from the germplasm population based on the GWAS results were planted in the plots with three replicates. Each plot comprised three 1.5 m rows along the minirhizotron pipes and were spaced 10 cm apart; each row had 15 holes with two seedlings per hole. Minirhizotron images were collected at booting and mid-grain fill by a CI-600 Roots Monitoring System (CID Inc., Camas, WA, USA) (Fig. 1G). For each minirhizotron pipe, eight images of 216 × 196 mm were collected and saved in tiff format. Total root length (TRL) was measured using WinRHIZO Tron MF image analysis software (Regent Instruments Inc., Quebec, Canada).

### Shoot trait phenotyping

All 323 wheat accessions were grown at Shunyi Experiment Station, Beijing (116°56′E, 40°23′N) in two growing seasons from October 2014 to June 2016, and at the Changping Experiment Station, Beijing (116°13′E, 40°13′N) in one growing season from October 2015 to June 2016. These two sites and the Institute of Crop Science Experimental Station had similar environmental conditions and soil types. The experimental fields were irrigated three times with 750 m^3^ ha^−1^: before winter, at booting and at flowering when the amount of rainfall was insufficient during each corresponding period (Fig. 2). Each experimental plot was 2 m in length with four rows and row spacing of 30 cm, 40 seeds per row. Field management was consistent with local practices for wheat production. Phenotypic measurements included plant height (PH), yield per plant (YPP) and canopy temperature (CT). Five healthy individuals randomly selected from each plot were used to investigate PH and YPP. PH was the distance between the top of the spike excluding awns and the stem base. CT was measured using an Optris LS infrared thermometer (IRT) at mid-grain fill when all accessions were about 2 weeks post-anthesis; in order to reduce environmental influences, all measurements were carried out from 11.30 to 13.30 h on calm, cloudless days by holding the IRT with a 30° angle at 20 cm above the canopy.

### Genotyping

Genomic DNA was extracted from seedling leaves according to the protocol of Ogbonnaya *et al.* (2001). A wheat 660K SNP Array designed by the Chinese Academy of Agricultural Sciences and synthesized by Affymetrix^®^ was applied to genotype all 323 wheat accessions. This array was developed from the transcriptome and genome sequence and consisted of 630 517 SNPs. The physical locations of SNPs were identified based on the IWGSC wheat genome sequence (IWGSC RefSeq v1.0). After removing nucleotide variations with missing rates ≥0.2 and minor allele frequency (<0.05), 395 675 SNPs were used for GWAS (Supplementary Data Fig. S2).

### Statistical analysis

Statistical analyses were performed using SPSS 19.0 software. Broad-sense heritability (*H*^2^) and a combined mixed linear model-based analysis of variance were calculated using the analysis of variance (ANOVA) procedure in IciMapping 4.1 software (Meng *et al.*, 2015). The overall phenotypic value used for GWAS were calculated as the best linear unbiased prediction (BLUP), which was based on a mixed linear model and performed using the function of ‘lmer’ in the R package lme4; the same method was used to calculate the overall performances of accessions to eliminate environment effects (C. Fang *et al.*, 2017).

The population structure of the 323 wheat accessions was assessed using 1800 SNP markers (at least 5 Mb apart and uniformly distributed across the genome) with the software STRUCTURE 2.3.4 based on admixture and correlated allele frequency models. The number of sub-populations (*k*) was set from 2 to 9. For each *k*, ten runs were performed separately; each run was carried out with 100 000 iterations and 100 000 burn-in periods. A Neighbor–Joining tree was constructed using the Megacc software on the basis of a distance matrix, using the whole-genome SNPs shared by all the accessions. Principal component analysis (PCA) was performed via GCTA software. Marker–trait relationships were analysed using both general linear models (GLMs) and mixed linear models (MLMs) in TASSEL 5.0 software,. Population structure was represented by the first five principal components, which were fitted as fixed effects; kinship (family relatedness) was used to define the random variables for the total genetic effects of the 323 accessions. Because the Bonferroni correction at the 0.05 level (0.05/395 675 = 1.26 × 10^–7^) was too conservative and could lead to false negatives (Zhao *et al.*, 2011; Gyawali *et al.*, 2016), a less stringent threshold of –log_10_(*P*) >4 was used to declare the significance of SNP–trait associations. Linkage disequilibrium (LD) was estimated as the squared allele frequency correlation (*r*^2^), for each SNP pair on the same chromosome, and *r*^2^ values were evaluated with the −*r*^2^ command in the software PLINK 1.9. An LD decay graph of each chromosome was plotted according to the average value of *r*^2^ of SNP pairs within 100 kb; step size was set to 100 kb. The pairwise *r*^2^ values were averaged across each sub-genome and the whole genome to get the LD delay graph of the corresponding genome. The extended region where LD between the significantly associated SNP and nearby SNPs decayed to *r*^2^ = 0.5 was regarded as the candidate region. For the same trait, each candidate region or overlapping candidate regions was categorized as an SAL; for different traits, the degree of linkage between each two SALs in the same chromosome was represented by their average LD using a previously reported method (C. Fang *et al.*, 2017), and an association network based on linkage between SALs was displayed using Cytoscape 3.6.1 software. Candidate genes were predicted using TriAnnot Pipeline 4.3.1. Gene annotation was performed by Wheat Zapper (Alnemer *et al.*, 2013). Protein sequence alignments were performed using DNAMAN 7.0 software.

## RESULTS

### Phenotypic analysis

The mean value of root depth increased 97.2 % from booting (57.4 cm) to mid-grain fill (113.2 cm), and the root dry weight increased 72.0 % from 0.25 to 0.43 g per plant during the same period. These results indicated that wheat roots rapidly elongated from the booting to mid-grain fill stages (Table 1). The coefficient of variation of root traits ranged from 12.4 % for root depth at booting to 30.2 % for root dry weight at mid-grain fill, showing a high degree of dispersion. The coefficient of variation of PH, YPP and CT is 20.2, 25.5 and 2.2 %, respectively, indicating that CT was a relatively stable shoot trait across accessions, which could be distinguished by small differences. The variations in PH, YPP and CT differed significantly among genotypes (G; *P* < 0.001), and among environments (E; *P* < 0.001); further, PH, YPP and CT of each genotype responded differently to environment (G × E, *P* < 0.05 or *P* < 0.01) (Table 1). Broad-sense heritabilities (*H*^2^) for all traits were moderate to high, ranging from 56.0 % for YPP to 94.6 % for PH, suggesting that these traits were suitable candidates for genetic analysis.

**Table 1.** Descriptive statistics, ANOVA results and broad-sense heritabilities estimated for root and shoot traits of 323 wheat accessions

Trait	Max	Min	Mean	s.d.	CV (%)	G	E	G × E	*H* ^2^ (%)
RDB (cm)	83.0	41.7	57.4	7.1	12.4	***	–	–	78.7
RDG (cm)	174.3	61.0	113.2	22.8	20.1	***	–	–	79.1
RDWB (g)	0.55	0.13	0.25	0.06	24.0	***	–	–	78.2
RDWG (g)	0.68	0.17	0.43	0.13	30.2	***	–	–	76.8
PH (cm)	138.1	61.3	97.3	19.7	20.2	***	***	*	94.6
YPP (g)	17.0	3.2	10.2	2.6	25.5	***	***	**	56.0
CT (°C)	28.5	24.5	26.7	0.6	2.2	***	***	**	67.9

RDB, root depth at booting stage; RDG, root depth at mid-grain fill stage; RDWB, root dry weight at booting stage, RDWG, root dry weight at mid-grain fill stage; PH, plant height; YPP, yield per plant; CT, canopy temperature. CV, coefficient of variation; G, genotype; E, environment; G × E, genotype × environment; *H*^2^, broad-sense heritability.

Significance level: *P < 0.05, **P < 0.01, ***P < 0.001.

**Fig. 3.**
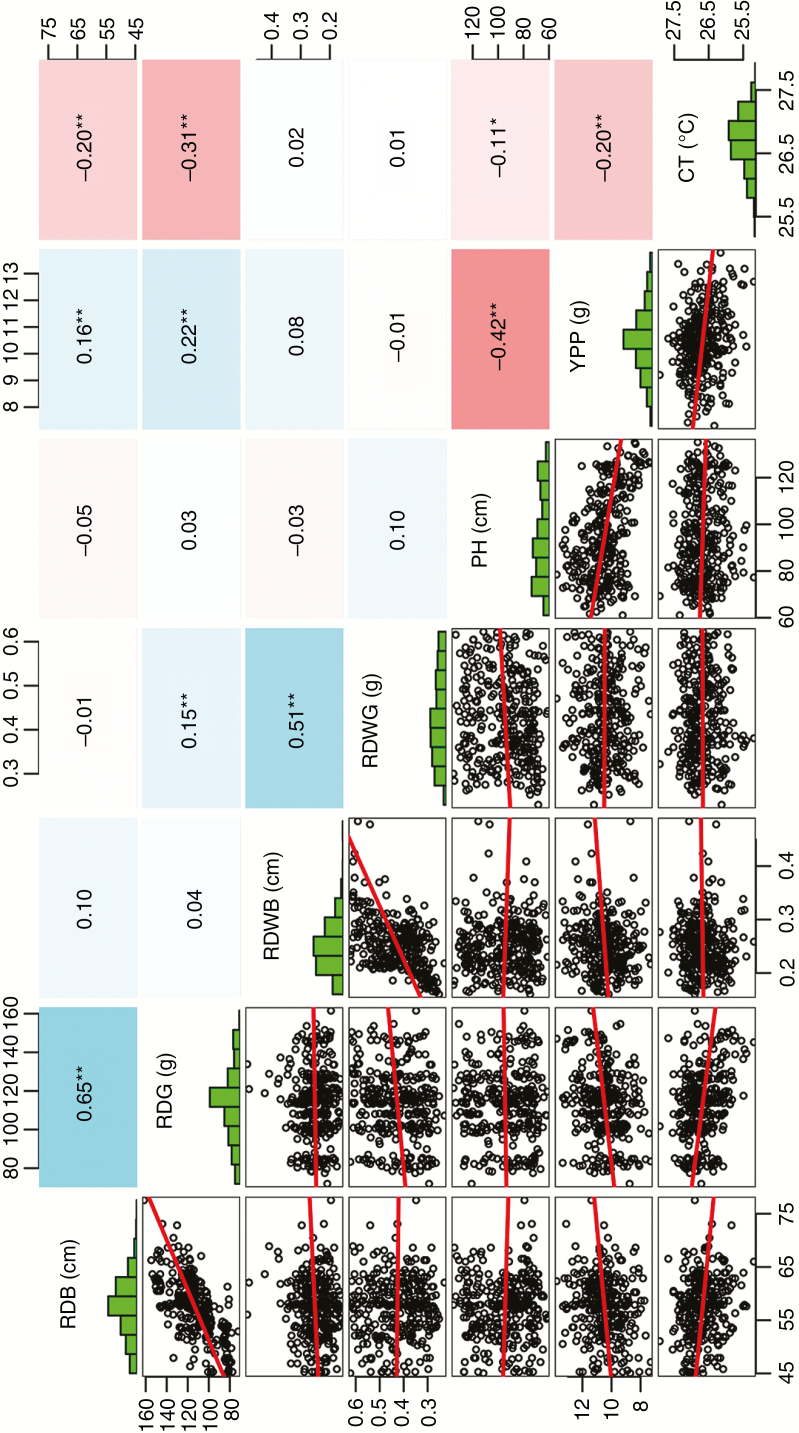
Correlation analyses and frequency distributions of root and shoot traits. The bottom left part of the matrix shows scatter plots of root and shoot traits; red lines indicate linear regressions. The upper right section shows correlation coefficients; colour shading indicates levels of correlation; and the diagonal shows histograms of different traits. RDB, root depth at booting; RDWB, root dry weight at booting; RDG, root depth at mid-grain fill; RDWG, root dry weight at mid-grain fill; PH, plant height; YPP, yield per plant; CT, canopy temperature; **P < 0.01.

Correlation analysis showed that the same root traits measured at different growth stages were positively correlated, with Pearson correlation coefficients of 0.65 for root depth and 0.51 for root dry weight. The only correlation between root depth and root dry weight was a significant but weak relationship of 0.15 at mid-grain fill (Fig. 3). Although root traits may not behave consistently in PVC tubes and field conditions, there were weak positive correlations between YPP and both root depth at booting stage (RDB, 0.16) and mid-grain fill stage (RDG, 0.22), and weak negative correlations between CT and both RDB (–0.20) and RDG (–0.31). No significant correlations were observed for PH and root traits, either for root dry weight or for shoot traits (Fig. 3).

**Fig. 4.**
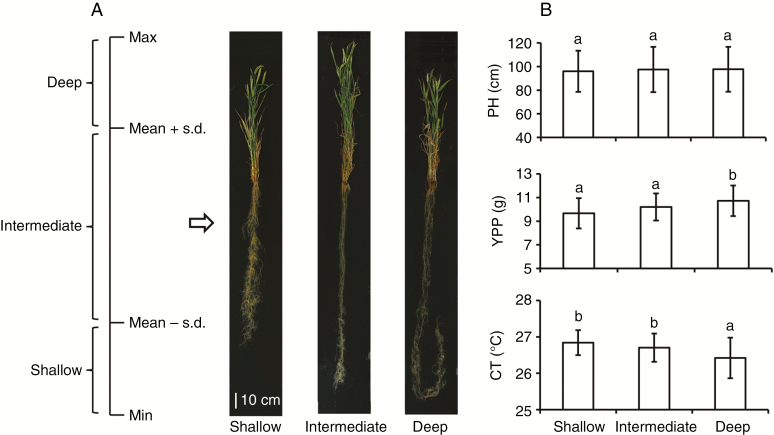
Root type classification at the mid-grain fill stage and comparison of shoot traits. (A) Roots of the 323 accessions were set into three types: deep (root depth > mean + s.d.), intermediate (mean + s.d. > root depth > mean – s.d.) and shallow (root depth < mean – s.d.). (B) The comparison of shoot traits of accessions with different root types. PH, plant height; YPP, yield per plant; CT, canopy temperature; different letters indicate the significant differences at *P* < 0.05; error bars denote the s.d.

To clarify further the effect of root depth on shoot traits, RDB and RDG of the 323 accessions were set into three types separately: deep (root depth > mean + s.d.), intermediate (mean + s.d. > root depth > mean – s.d.) and shallow (root depth < mean – s.d) (Fig. 4A; Supplementary Data Fig. S3A). The average PH of accessions with different root types were not significantly different, whereas deep root accessions had lower CT and higher YPP than shallow root accessions (Fig. 4B; Supplementary Data Fig. S3B). For instance, the average YPP of accessions with deep roots at mid-grain fill stage was 11.0 g, 9.0 % higher than those with shallow roots (10.1 g); the average CT of accessions with deep roots at mid-grain fill was 26.3 °C, significantly lower than those with shallow roots (26.8 °C) (Fig. 4B).

### Population structure and LD analysis

**Fig. 5.**
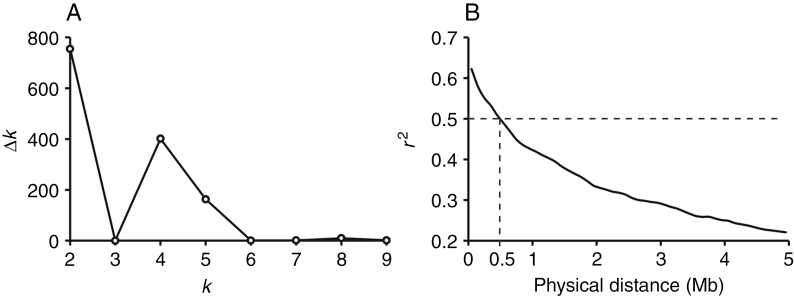
Population structure and linkage disequilibrium (LD) analysis. (A) Plot of Δ*k* against putative *k* ranging from 2 to 9. (B) Decay of LD for the whole genome; horizontal and vertical dashed lines indicates that LD declined to 0.5 at about 0.5 Mb.

Diversity panels often represent population structure that generates spurious associations between the phenotypic values and unlinked markers. Therefore, better understanding of population structure is the prerequisite to conducting successful association mapping. The most likely number of sub-populations was two (*k* = 2) according to the methodology of Evanno (Evanno *et al.*, 2005) (Fig. 5A). This result was confirmed by PCA based on standardized covariance of genetic distances of SNP markers, where the top two principal components explained 6.8 and 5.1 %, respectively, of the genetic variance in the population and separated the two sub-populations (Supplementary Data Fig. S4A); the Neighbor–Joining tree also suggested that the 323 accessions could be classified into two main clades (Supplementary Data Fig. S4B), which were consistent with the results mentioned above. The extent of LD decay determines the mapping resolution and power. The LD among SNPs was calculated for each chromosome, sub-genome and at the whole-genome level. The LD (indicated by *r*^2^) dropped to 0.5 at 0.5 Mb for the whole genome (Fig. 5B) but with variations among different sub-genomes. The LD extent in the A genome was 0.35 Mb, similar to that of the D genome (0.25 Mb), but much smaller than that of the B genome (0.75 Mb) (Supplementary Data Fig. S5).

### Whole-genome screening for significantly associated loci

A total of 870 SALs were identified by GLM, 326, 246, 82, 28, 41, 45 and 102 of which were associated with PH, YPP, CT, RDB, RDG, RDWB and RDWG, respectively (Supplementary Data Table S2). However, only 93 SALs were detected by MLM (Supplementary Data Table S3), which is far less than by GLM, hence showing the latter’s better effectiveness at reducing false positives. In detail, 25 SALs distributed on chromosomes 1D, 2B, 2D, 3A, 4A, 4D, 5A, 5B, 6D, 7A, 7B and 7D were associated with PH, and the phenotypic variation explained by each peak SNP ranged from 4.7 to 18.7 %; 16 SALs distributed on chromosomes 1A, 2A, 2B, 2D, 3B, 3D, 5A, 5B, 6A, 6B and 7A were associated with YPP, and the phenotypic variation explained by each peak SNP ranged from 4.7 to 6.9 %; 16 SALs distributed on 15 chromosomes (except for 1D, 3A, 3B, 3D, 5B and 7D) were associated with CT, and the phenotypic variation explained by each peak SNP ranged from 4.8 to 16.7 %; 15 and six SALs distributed on ten chromosomes (2A, 2B, 2D, 3D, 4A, 4B, 5B, 6A, 6B and 6D) and six chromosomes (2A, 2B, 2D, 5A, 6A and 6D) were associated with root depth at booting and mid-grain fill stage, respectively, and the phenotypic variation explained by each peak SNP ranged from 4.8 to 7.8 %, and from 5.1 to 6. 5%; and ten and five SALs distributed on nine chromosomes (1B, 2B, 2D, 4B, 4D, 6A, 6B, 6D and 7A) and four chromosomes (1B, 2A, 2B and 4B) were associated with root dry weight at booting and mid-grain fill stage, respectively, and the phenotypic variation explained by each peak SNP ranged from 4.8 to 7.8 %, and from 5.0 to 5.9 %.

**Fig. 6.**
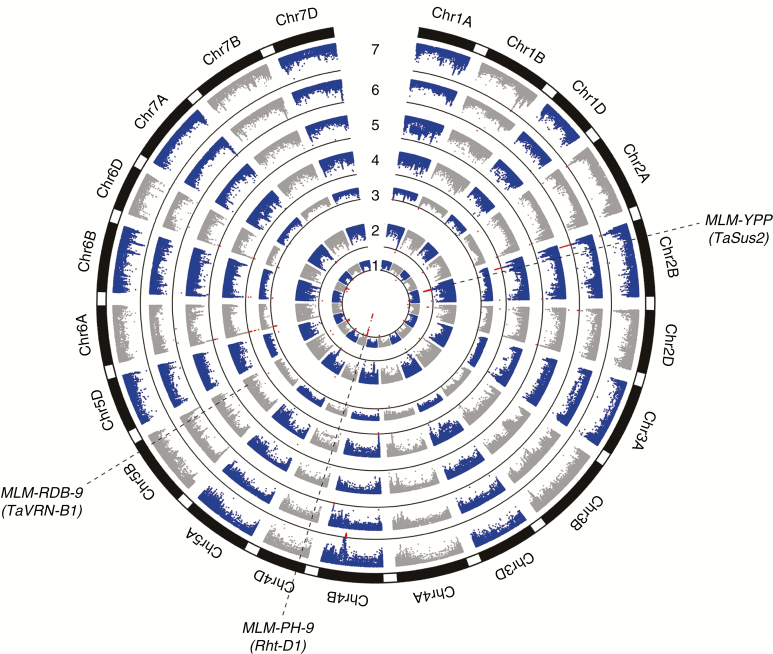
GWAS results for root and shoot traits based on the mixed linear model. Numbers iindicate PH (1, plant height), YPP (2, grain yield per plant), CT (3, canopy temperature), RDB (4, root depth at booting stage), RDG (5, root depth at mid-grain fill stage), RDWB (6, root dry weight at booting stage) and RDWG (7, root dry weight at grain fill stage). Red dots represent significantly associated SNPs, black circular lines indicate the threshold value (*P* < 1 × 10^–4^), and the locations of significantly associated loci overlapping reported genes (within parentheses) are shown as black dashed lines.

Both the GLM and the MLM consistently detected strong signal linked to previously reported related genes. For instance, *GLM-PH-131* and *MLM-PH-9* located on chromosome 4D overlapped with the green revolution gene *Rht-D1* (Peng *et al.*, 1999); *GLM-YPP-42* and *MLM-YPP-3* located on chromosome 2B overlapped with *TaSus2-2B*, a wheat sucrose synthase 2 gene associated with yield traits (Jiang *et al.*, 2011); and *GLM-RDB-21* and *MLM-RDB-9* located on chromosome 5B overlapped with *TaVRN-B1*, a key regulator of shoot and root architecture (Voss-Fels *et al.*, 2018) (Fig. 6; Supplementary Data Fig. S6). Interestingly, several SALs near other PH-controlling genes such as *Rht-B1* on 4B (Peng *et al.*, 1999) and *Rht18* on 6A (Ford *et al.*, 2018), and other yield-controlling genes such as *TaGW2* on 6A (Bednarek *et al.*, 2012), *TaSPL21-B* on 6B and *TaSPL21-D* on 6D (Zhang *et al.*, 2017), were only detected by the GLM (Supplementary Data Fig. S6). This suggests that the mixed model may overcompensate for population structure and relatedness, leading to false negatives.

### Genetic network of loci associated with root and shoot traits

**Fig. 7.**
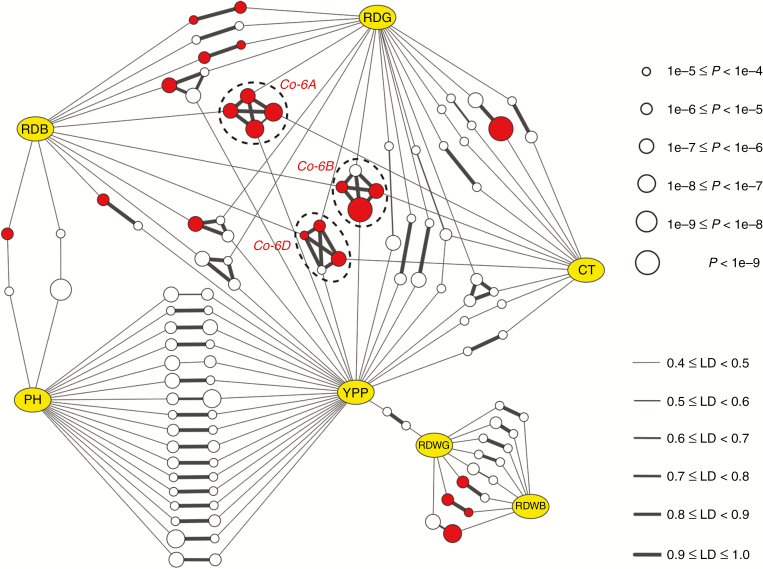
Association networks across different root and shoot traits in wheat. The yellow ellipses represent traits, and the white and red circles represent the SALs responsible, which are detected by GLM and both models, respectively. Only the SALs associated with at least two traits are displayed; the size of the circle indicates the probability of associations. PH, plant height; YPP, yield per plant; CT, canopy temperature; RDB, RDWB: root depth, root dry weight at booting stage; RDG, RDWG: root depth, root dry weight at mid-grain fill stage. The edges between the SALs from different traits are linked by linkage disequilibrium (LD). Only the edges with an average LD ≥ 0.4 are displayed; the thickness of lines indicates the degree of LD. The overlapped SALs (*Co-6A*, *Co-6B* and *Co-6D*) that not only control YPP and CT, but also exhibited stable associations with root depth at different growth periods are highlighted by the dotted black circles.

To dissect the genetic architecture of the correlations across different traits and identify hub SALs, we constructed an association network based on LD between each two overlapping SALs controlling different traits (LD values ≥0.4 were considered to be linked) (Fig. 7). Considering the false negatives of MLM mentioned above, we observed that there are few overlaps between SALs for different traits and realized that it might be too conservative. Therefore, the GWAS results of GLM and MLM were combined to construct the association network. We observed that 17 SALs controlling PH linked to SALs for YPP; the LD values of 14 pairs of these linked SALs were >0.8 (Fig. 7). We also found 11 SALs controlling root depth (RDB or/and RDG) linked to SALs for YPP, and the LD values of ten pairs were >0.9; more importantly, four of them also linked to SALs for CT (Fig. 7). However, we noted that only two SALs controlling RDB weakly linked to PH (LD <0.5), and SALs controlling root dry weight (RDWB or/and RDWG) tended to be independent and barely linked to SALs controlling other traits (Fig. 7). These results are consistent with the correlation pattern of the traits (Fig. 3), suggesting that the linkage or pleiotropy of hub SALs largely determines the relationships among traits. Additionally, it is noteworthy that there are three genomic regions, i.e. 6A (base pairs 15 202 326–16 883 226), 6B (base pairs 26 133 579–27 905 077) and 6D (base pairs 14 009 924–16 166 519), detected by both GLM and MLM, not only controlling YPP and CT, but exhibiting stable associations with root depth at different growth periods. For further experiments, we named these co-located SALs as *Co-6A*, *Co-6B* and *Co-6D*, respectively (Fig. 7; Supplementary Data Fig. S7).

**Fig. 8.**
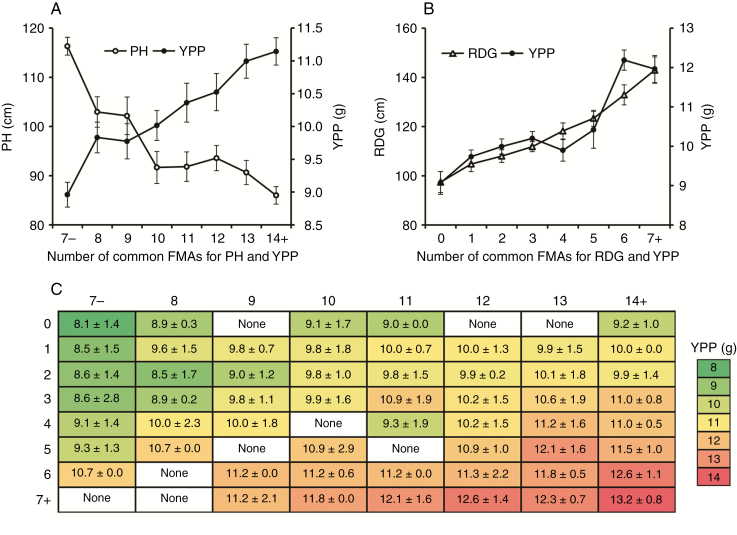
The combined effect of common FMAs in SALs. (A) YPP increased and PH decreased along with the combination of FMAs associated with YPP and PH. (B) YPP and RDG increased along with the combination of FMAs associated with YPP and RDG. (C) The heatmap of YPP based on the combination of FMAs associated with YPP and PH (horizontal) and FMAs associated with YPP and RDG (vertical). YPP, yield per plant; PH, plant height; RDG, root depth at mid-grain fill stage; error bars in (A) and (B) and error values in (C) denote the s.e.

We further analysed the combined effect of hub SALs (SALs displayed in Fig. 7) based on the peak SNPs (SNPs with a minimum *P*-value in GWAS). Each peak SNP has two types of marker alleles, one is responsible for higher yield and is called the favourable marker allele (FMA). We observed that YPP increased and PH decreased along with the combination of FMAs associated with YPP and PH simultaneously (Fig. 8A); moreover, both YPP and RDG increased along with the combination of FMAs associated with YPP and RDG simultaneously (Fig. 8B). These results suggested that the number of FMAs for dwarf and deep rooting was related to high yield. FMAs controlling at least two traits were defined as common FMAs. In addition, we observed that the yield gain is limited by merely combining the common FMAs associated with YPP and PH, and this obstacle may be overcome by further combining common FMAs associated with YPP and RDG; for instance, the YPP of accessions without RDG-associated FMAs increased from 8.1 to 9.2 g by combining PH-associated FMAs alone, which can be further increased to 13.2 g by combining RDG-associated FMAs (Fig. 8C).

### Distribution of common FMAs in cultivars of different eras

**Fig. 9.**
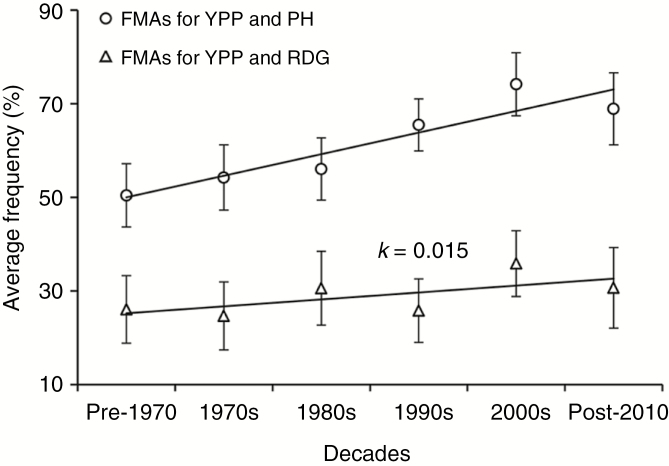
Average frequency of common FMAs associated with YPP and PH, and FMAs associated with YPP and RDG. PH, plant height; YPP, yield per plant; RDG, root depth at mid-grain fill stage; *k*, the slope of linear regressions.

Since the SALs controlling PH and root depth are crucial for wheat improvement, we further analysed the breeding selection on common FMAs controlling YPP and PH simultaneously, and common FMAs for YPP and RDG. Based on year of release, all accessions were chronologically classified into six groups: group I (pre-1970), group II (1970s), group III (1980s), group IV (1990s), group V (2000s) and group VI (post-2010). We observed that the average frequency (FMAs/total marker alleles ratio for each peak SNP) of common FMAs associated with YPP and PH ranged from 50.4 to 68.9 %, higher than that of common FMAs associated with YPP and RDG (ranging from 26.0 to 35.8 %) (Fig. 9). In addition, the average frequency of common FMAs controlling different traits all showed increasing trends during past decades, but the increase rate (*k*, indicated by the slope of linear regression, generated with the decades and average FMA frequency) for common FMAs associated with YPP and PH was 0.046, three times that of FMAs controlling YPP and RDG (0.015) (Fig. 9), suggesting that the selection pressure and utilization for common FMAs controlling YPP and RDG are weaker compared with those for YPP and PH.

### Causal gene prediction for hub SALs

We focused on three loci *Co-6A*, *Co-6B* and *Co-6D*, because these not only control YPP and CT, but also exhibited stable associations with root depth at different growth stages. The peak SNPs detected by MLM for different traits in each of the three loci are the same or highly linked. To be specific, for *Co-6A*, AX-110520701 is the shared peak SNP for RDB and RDG, AX-111048945 is the peak SNP for YPP and CT, and the LD between these two SNPs is 0.88; for *Co-6B*, AX-94940485 is the shared peak SNP for YPP, CT and RDB; for *Co-6D*, AX-108848017 is the peak SNP for CT, AX-94788899 is the peak SNP for RDB and RDG, and the LD between these two SNPs is 1.00. These results implied that the causal genes for different traits in *Co-6A*, *Co-6B* and *Co-6D* are the same or highly linked. Using the TriAnnot Pipeline, genes in the three loci were predicted. Interestingly, the peak SNPs in *Co-6A*, *Co-6B* and *Co-6D* were all near to predicted transporter genes, in particular for AX-94940485 on *Co-6B*, which were inside the predicted transporter genes, and it is noteworthy that 13 transporter genes were within each of the three loci (Supplementary Data Fig. S8). Amino acid sequence alignments showed that the predicted transporter genes had high degrees of similarity with wheat high-affinity nitrate transporters genes (*TaNRT2.1*, *TaNRT2.2* and *TaNRT2.3*), with sequence identities ranging from 80.2 to 100 % (Supplementary Data Table S4), thus suggesting that they belonged to the *NRT2* gene family.

**Fig. 10.**
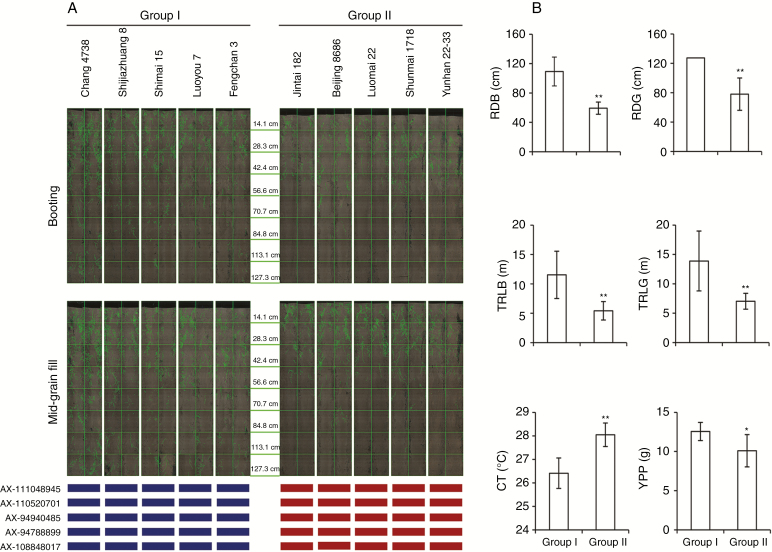
Comparison of root and shoot traits in the field between accessions with different marker alleles at peak SNPs in *Co-6A*, *Co-6B* and *Co-6D* by the minirhizotron system. (A) Minirhizotron scanning images. Group I consisted of five accessions with deep-root FMAs of all peak SNPs in *Co-6A*, *Co-6B* and *Co-6D*; Group II consisted of five accessions with alternative shallow root marker alleles in these peak SNPs. Blue rectangles indicate deep-root FMAs, red rectangles represent alternative marker alleles. (B) Comparisons of root depth at booting stage (RDB) and mid-grain fill stage (RDG), total root length at booting stage (TRLB) and mid-grain fill stage, canopy temperature (CT) and yield per plant (YPP) between Group I and Group II. *, ** significant differences at the 0.05 and 0.01 level, respectively; error bars denote the s.d.

In order to investigate further the causal functions of genes in *Co-6A*, *Co-6B* and *Co-6D*, the root traits of two groups of selected accessions were measured in the field using a minirhizotron system. Group I consisted of five accessions with deep-root FMAs of all peak SNPs (detected by MLM) in *Co-6A*, *Co-6B* and *Co-6D*. Group II consisted of five accessions with alternative shallow root marker alleles in each peak SNP. The average root depths in PVC pipe culture of Group I accessions at booting and mid-grain fill were 68.7 and 147.3 cm, respectively, compared with Group II with corresponding values of 48.1 and 73.9 cm. Scanning by the minirhizotron system showed that the root depth in the field was significantly correlated with root depth in the PVC pipe culture (Pearson correlation coefficient was 0.797 at the booting stage), and average root depths in the field of Group I were 109.3 cm at booting and 127.3 cm at mid-grain fill (maximum depth of the minirhizotron was 127.3 cm), significantly deeper than those of Group II (59.4 and 78.1 cm, respectively) (Fig. 10). Furthermore, the average total root lengths of Group I were 11.5 and 13.9 m at booting and mid-grain fill, far longer than those of Group II at 5.4 and 7.0 m, respectively. The average canopy temperature of Group I was 26.4 °C at mid-grain fill, significantly lower than that of Group II of 28.0 °C. The average yield per plant of Group I was 12.5 g, significantly higher than that of Group II of 10.1 g (Fig. 10B). These results suggested that the specific marker alleles in the peak SNPs in *Co-6A*, *Co-6B* and *Co-6D* could predict root depth in the field, and the causal genes influenced root length, hence the association among root depth and shoot traits in wheat.

## DISCUSSION

### Phenotyping methods for root traits

Measurement of root traits at the flowering and grain development growth stages is very difficult. There are two major phenotyping methods, namely destructive methods (monoliths, soil cores and mesh bags) (Sharma *et al.*, 2011; Trachsel *et al.*, 2011; Lou *et al.*, 2015) and non-destructive methods (optical scanners, minirhizotrons and electrical capacitance) (Dannoura *et al.*, 2008; Amato *et al.*, 2009; Ao *et al.*, 2010). Considering accuracy, workload, cost and throughput – each one has merits and demerits (Zhu *et al.*, 2011) – we grew wheat in PVC pipes to detect root traits for GWAS, mainly because this method can provide a similar growth environment in soil for all accessions, and root depth measured before washing accurately reflected the variation among accessions. In addition, roots with high integrity can be acquired after careful washing. For functional verification of causal genes, we used the minirhizotron system because it dynamically recorded root traits *in situ*, permitting observations on root traits at different growth stages in the field.

### Phenotypic variation of wheat roots

It was previously reported that wheat roots grow rapidly during the seedling period, slowly down during tillering and rapidly expand until flowering, after which root growth is slow (Tennant, 1976). Our study found that root depth and root dry weight rapidly increased by 97.2 and 72.0 %, respectively, from booting to mid-grain fill. We thus speculate that the booting to flowering period is crucial for wheat root elongation, and our data confirm the importance of root studies at late growth stages. Only a weak positive correlation was detected between maximum root depth and root dry weight at mid-grain fill, suggesting that the distribution of roots in soil layers is quite variable among accessions. This difference largely determines the ability to absorb nutrients and water from soil. For example, phosphorus is mainly located in the shallow soil layers, whereas nitrogen and water are frequently found in the deep profile, especially at the late growth stages (Rogers and Benfey, 2015). These temporal and spatial phenotypic variations highlight the importance of multi-perspective thinking in root research.

### GWAS for root and shoot traits

Genome-wide association studies have many advantages over the traditional linkage mapping including use of natural germplasm and increased QTL resolution and allele coverage (Lou *et al.*, 2015; Sun *et al.*, 2016). However, due to the genome complexity, the application of GWAS in wheat lags behind when compared with other crop species such as rice and maize (Wang *et al.*, 2016; Yano *et al.*, 2016). In the present study, a Wheat 660K SNP Array based on the recently released IWGSC wheat genome sequence (IWGSC RefSeq v1.0) was used to detect genotypes for GWAS. A total of 93 SALs were identified based on MLM; some of them overlapped with functional genes such as *Rht-D1*, *TaSus2-2B* and *TaVRN-B1* (Peng *et al.*, 1999; Jiang *et al.*, 2011; Voss-Fels *et al.*, 2018), some of them located on the same chromosome as the reported QTLs controlling corresponding traits (Pinto *et al.*, 2010; Mason *et al.*, 2011; Ren *et al.*, 2012; Liu *et al.*, 2013; Rebetzke *et al.*, 2013; Cane *et al.*, 2014; Mondal *et al.*, 2015*a*; Ayalew *et al.*, 2017; Xie *et al.*, 2017), but most of them have not been previously reported to the best of our knowledge. We confirmed the physical intervals of these SALs on the respective chromosomes by LD analysis (Supplementary Data Table S3). These results enrich the genetic information and improve the accuracy of genetic mapping of root and shoot traits in wheat. In addition, a total of 870 SALs were detected based on GLM, which is far more than by MLM. Notably, strong signals linked to more functional genes, i.e. *Rht-B1*, *Rht18*, *TaGW2*, *TaSPL21-B* and *TaSPL21-D*, were detected (Peng *et al.*, 1999; Bednarek *et al.*, 2012; Zhang *et al.*, 2017; Ford *et al.*, 2018). This suggests that MLM may overcompensate for population structure and relatedness, which was also reported in previous studies (Zhao *et al.*, 2011; Gyawali *et al.*, 2016). Therefore, we used both GLM and MLM to identify SALs for further exploratory analysis such as association network construction.

### Relationship among root and shoot traits

In the present study, no significant correlation was observed among plant height and root traits, which is consistent with the conclusion reported by other wheat researchers and also supported by previous reports in other crops (Serraj *et al.*, 2004; Sanguineti *et al.*, 2007; Narayanan *et al.*, 2014). However, the *Rht* genes controlling dwarfism were reported in other studies to have pleiotropic effect on root traits (Wojciechowski *et al.*, 2009; Li *et al.*, 2011; Bai *et al.*, 2013), but most of these reports were focused on root traits at the seedling stage, whereas for studies focused on later growth stages, no significant difference was observed between *Rht* mutants and wild types (Narayanan and Prasad, 2014). Correspondingly, we detected only two SALs controlling both RDB and PH, but none of them overlapped with *Rht* genes. Taken together, our results suggest that the proposed relationships between root traits and plant height, and the function of dwarfing genes on root system regulation must be viewed with caution, especially for late growth stages. On the other hand, root depth at both booting and mid-grain stages showed significant positive correlations with YPP, and negative correlations with CT. For the mid-grain fill stage, we observed that the average YPP of accessions with deep roots is 9.0 % higher than that of those with shallow roots, and the average CT differed by 0.5 °C. Moreover, among 11 SALs controlling root depth which linked to SALs controlling YPP, four of them linked to CT as well, and the LD was >0.9 for each linked pair. Therefore, the significant phenotypic correlation and closely linked SALs observed in our study provide a genetic basis for using root depth as a beneficial trait for wheat improvement. In addition, the soil water content of the deep soil layer is higher than that of the shallow layer in our experimental fields (Fig. 2), suggesting that the advantages for grain yield of deep-rooting cultivars may partly be due to the absorption of deep soil water to maintain stomatal opening for photosynthesis, reflected in cooler canopies.

### Analysis of breeding selection and combined effect of SALs

Plant breeders generally assume that direct selection for yield will indirectly select varieties with the optimum root system for delivering the highest yields. For root depth, we observed that the frequency of FMAs in each cultivar showed an increasing trend during past decades, suggesting that breeders’ selection had positively favoured these genomic regions in wheat breeding. However, we also observed that the increased rate for common FMAs associated with YPP and PH was 0.046, higher than that of FMAs controlling YPP and RDG (0.015) (Fig. 9). It may be because lodging is positively correlated with PH, and short plants are more tolerant of lodging (Navabi *et al.*, 2006); as a consequence, plant height has been selected as a direct target in wheat breeding. On the other hand, it is known that heritable covariation adds to the complexity for breeding; for instance, it is hard to improve grain yield and quality simultaneously, since they are tightly integrated in a negative correlation (Klingenberg, 2008). Encouragingly, no significant correlation was observed among root traits and plant height as mentioned above; moreover, our results showed that grain yield per plant was significantly higher in genotypes which combined FMAs for root depth and plant height, suggesting that shorter plant height and deep roots are suitable traits for pyramiding target alleles by molecular marker-assisted breeding. Taken together, our results highlight that natural diversity of the root system is an untapped pool of useful genetic variation, and more direct selection for speciﬁc root architecture traits could be beneficial to enhance wheat production.

### Hub SALs for root and shoot traits

The hub SALs identified are expected to possess functional genes with application value (Guo *et al.*, 2018). In the present study, we identified three hub SALs (*Co-6A*, *Co-6B* and *Co-6D*) controlling RDB, RDG, CT and YPP. Using a minirhizotron system, we found that total root length of accessions with favourable marker alleles of peak SNPs at *Co-6A*, *Co-6B* and *Co-6D* were much longer than those of accessions with alternative marker alleles in these peak SNPs, suggesting that the causal genes at these SALs were associated with root elongation and account for differences in root depth; hence the association with canopy temperature and grain yield. Interestingly, 13 *NRT2* genes were located in each of the SALs and were close to the peak SNPs. The *NRT2* gene family encodes proteins that were identified as components of the homological *AtNRT2.1* high-affinity transport system, which is expressed mainly in roots. In rice, upregulated expression of *OsNRT2.1* improves nitrogen utilization efficiency and yield (Chen *et al.*, 2016); and knockout of *OsNRT2.4* resulted in a 20–40 % decrease of total later root length (Wei *et al.*, 2018). However, few studies in wheat have focused on the function of *NRT2* genes. Our results pinpoint the significance of *NRT2* genes in wheat improvement. Work is underway to verify the relationship and to elucidate the mechanism of the effectg *NRT2* genes on root growth in wheat, and thus the relationship among root traits and shoot performance.

## SUPPLEMENTARY DATA

Supplementary data are available online at https://academic.oup.com/aob and consist of the following. Table S1: information on 323 wheat accessions. Table S2: summary of the significantly associated loci detected by GLM. Table S3: summary of the significantly associated loci detected by MLM. Table S4: protein sequence similarity between reported wheat *NRT2* genes and predicted *NRT2* genes in *Co-6A*, *Co-6B* and *Co-6D.* Figure S1: frequency histogram of days to flowering for 323 winter wheat accessions. Figure S2: the density of ﬁltered SNPs used for GWAS. Figure S3: root type classification at the booting stage and comparison of shoot traits. Figure S4: the population structure of the 323 wheat accessions. Figure S5: decay of linkage disequilibrium for each chromosome and sub-genome. Figure S6: GWAS results for root and shoot traits based on the general linear model. Figure S7: associations between polymorphisms within hub SALs and the phenotypic variations, canopy temperature, root depth at booting stage and root depth at mid-grain fill stage, based on the mixed linear model. Figure S8: physical location of peak SNPs and predicted *NRT2* genes within *Co-6A*, *Co-6B* and *Co-6D*.

## Supplementary Material

mcz041_Suppl_Supplementary_Figure-S1

mcz041_Suppl_Supplementary_Figure-S2

mcz041_Suppl_Supplementary_Figure-S3

mcz041_Suppl_Supplementary_Figure-S4

mcz041_Suppl_Supplementary_Figure-S5

mcz041_Suppl_Supplementary_Figure-S6

mcz041_Suppl_Supplementary_Figure-S7

mcz041_Suppl_Supplementary_Figure-S8

mcz041_Suppl_Supplementary_Table-S1

mcz041_Suppl_Supplementary_Table-S2

mcz041_Suppl_Supplementary_Table-S3

mcz041_Suppl_Supplementary_Table-S4
