# Supplementary figures and images for: Genome-wide association study reveals genomic regions controlling root and shoot traits at late growth stages in wheat
Source: Ann Bot. 2019 Apr 9;124(6):993–1006. doi: 10.1093/aob/mcz041 (PMC6881226; doi:10.1093/aob/mcz041)

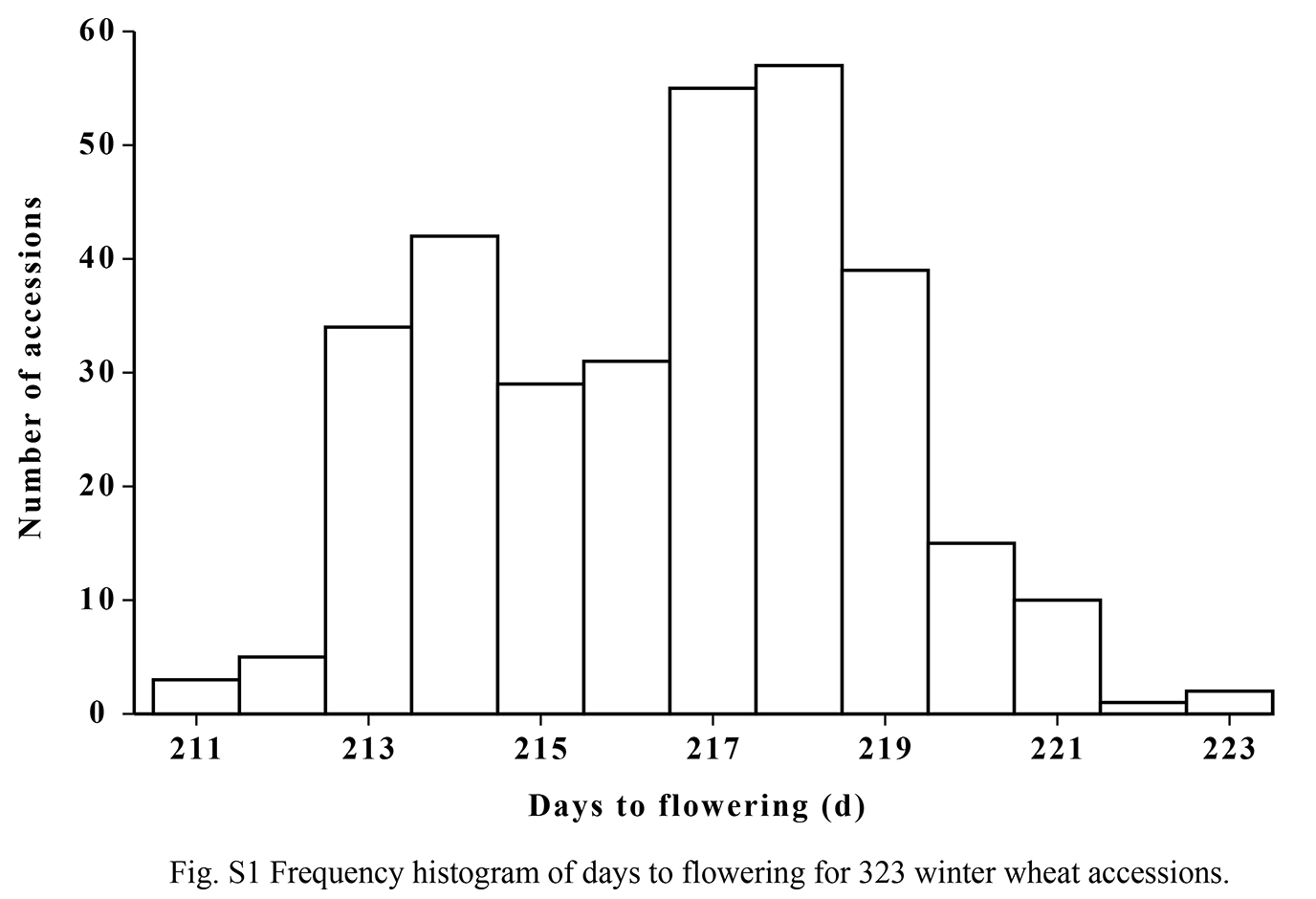

Supplement: mcz041_Suppl_Supplementary_Figure-S1 [file mcz041_suppl_supplementary_figure-s1.png]

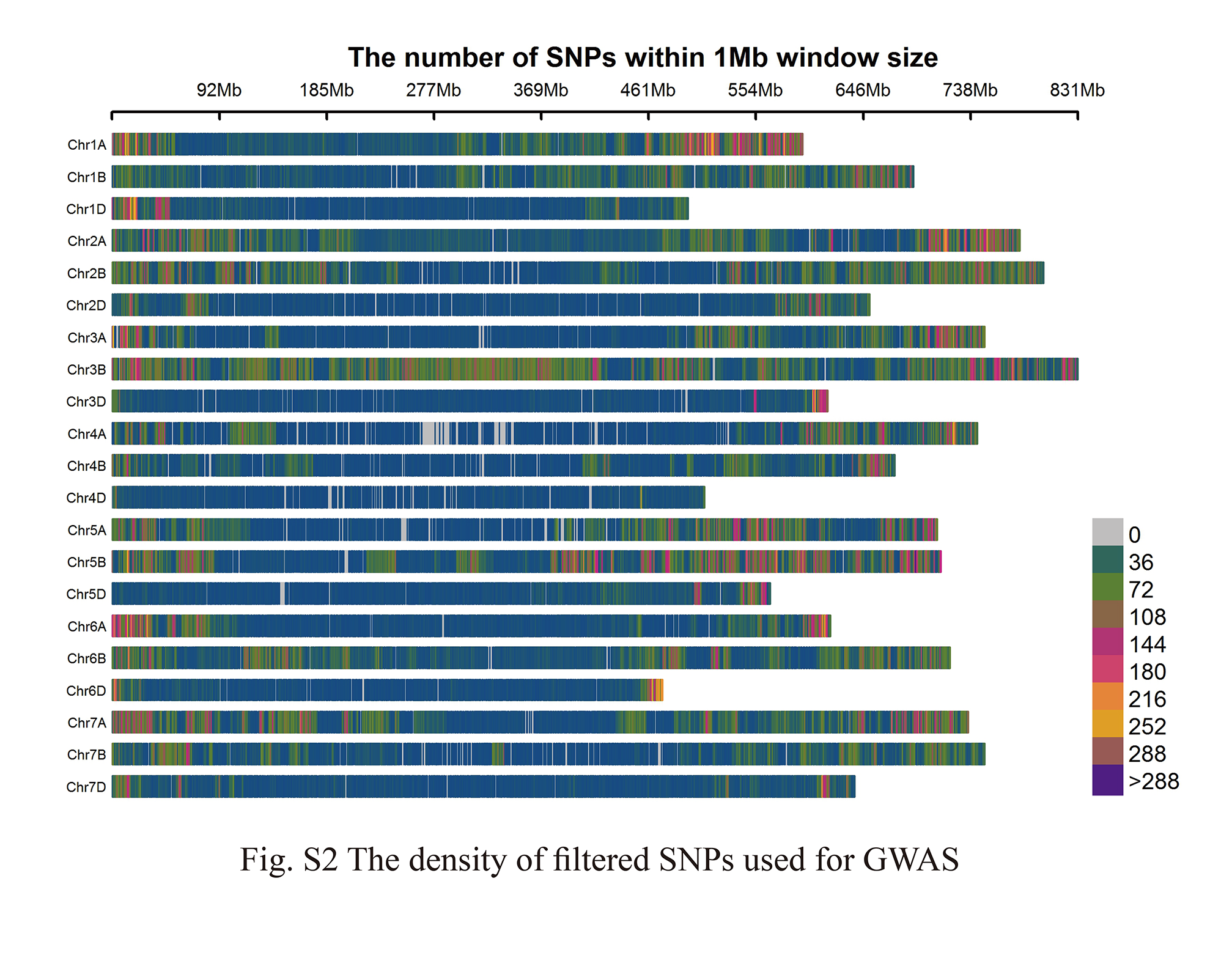

Supplement: mcz041_Suppl_Supplementary_Figure-S2 [file mcz041_suppl_supplementary_figure-s2.png]

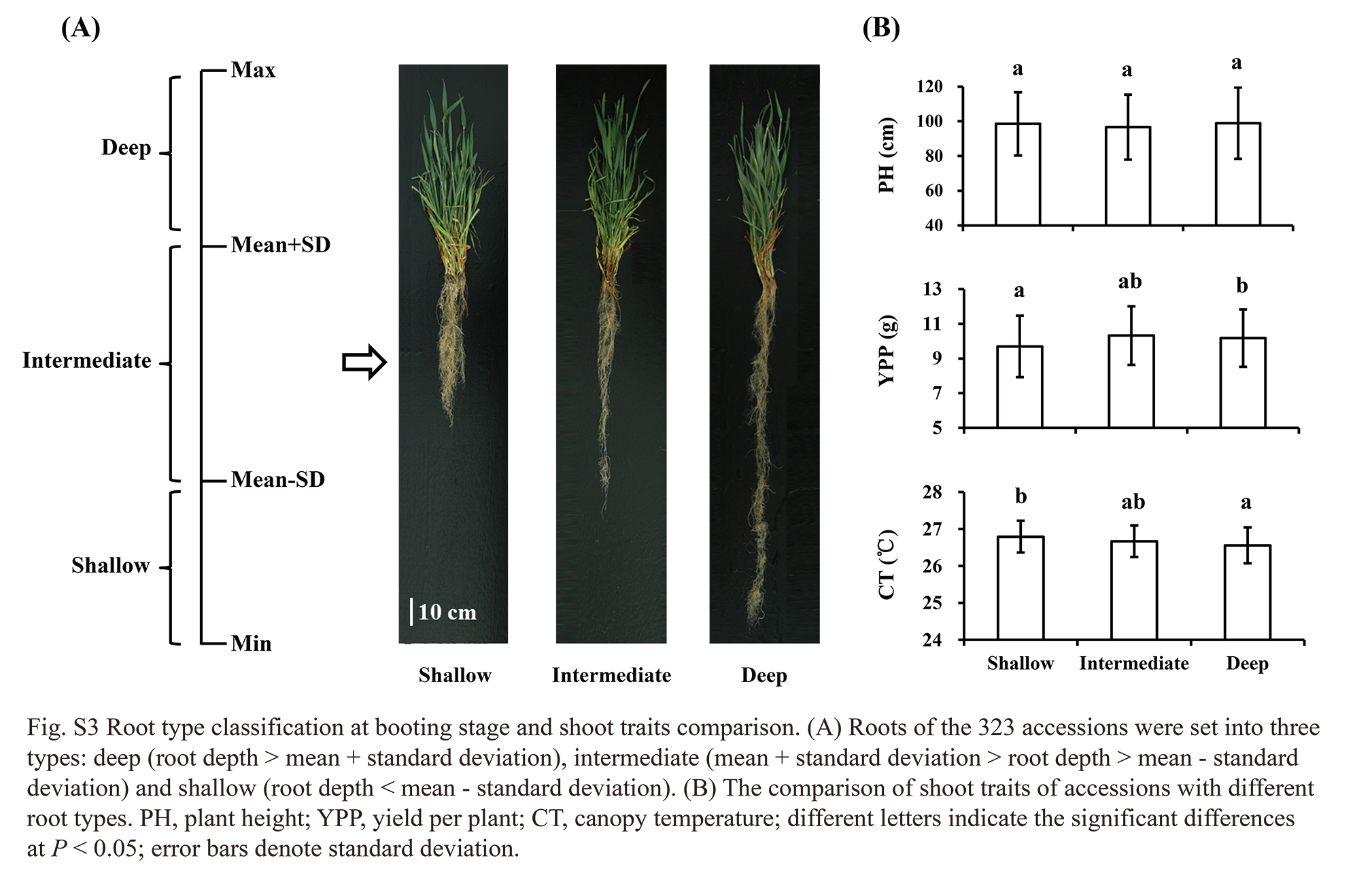

Supplement: mcz041_Suppl_Supplementary_Figure-S3 [file mcz041_suppl_supplementary_figure-s3.png]

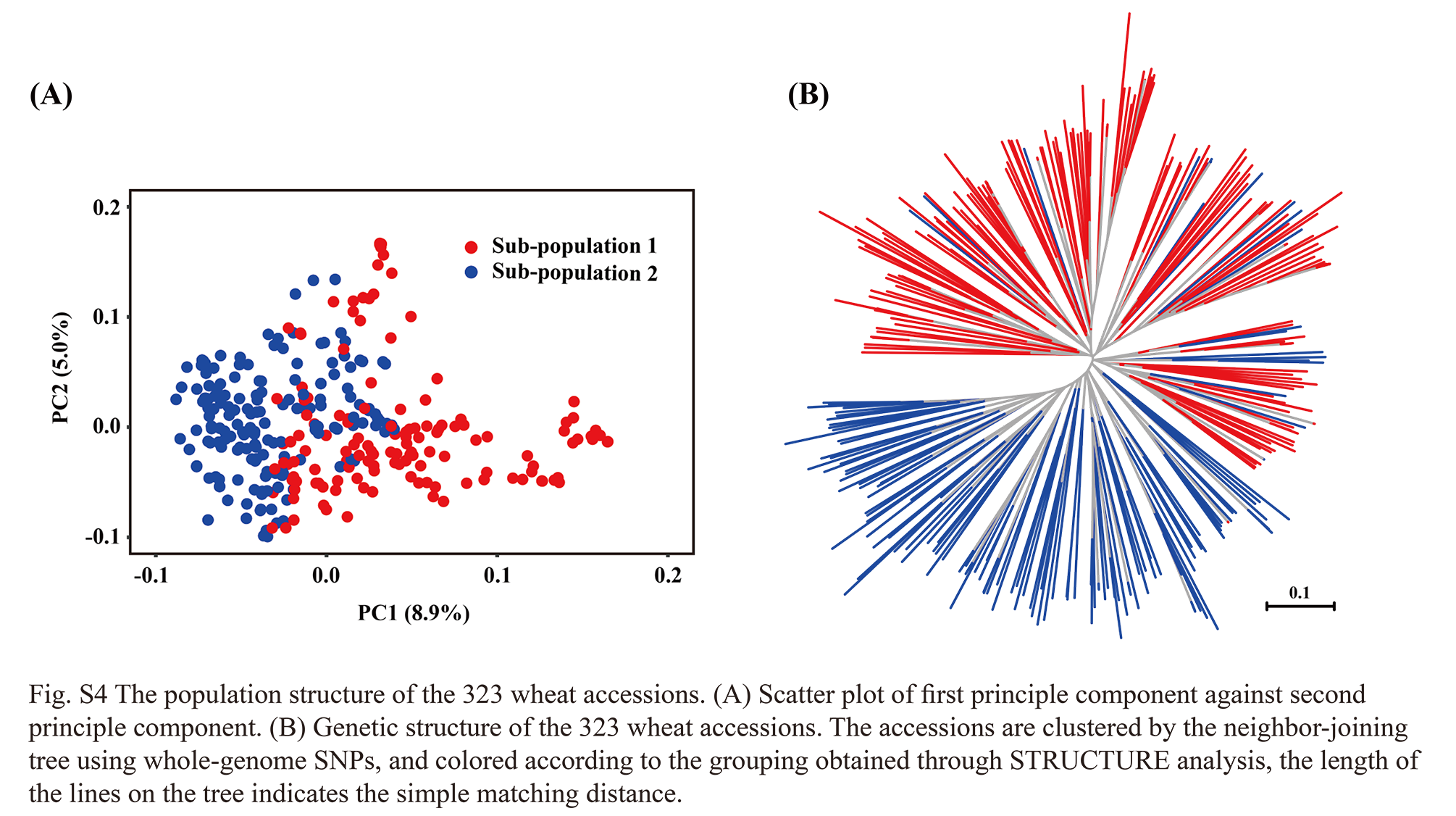

Supplement: mcz041_Suppl_Supplementary_Figure-S4 [file mcz041_suppl_supplementary_figure-s4.png]

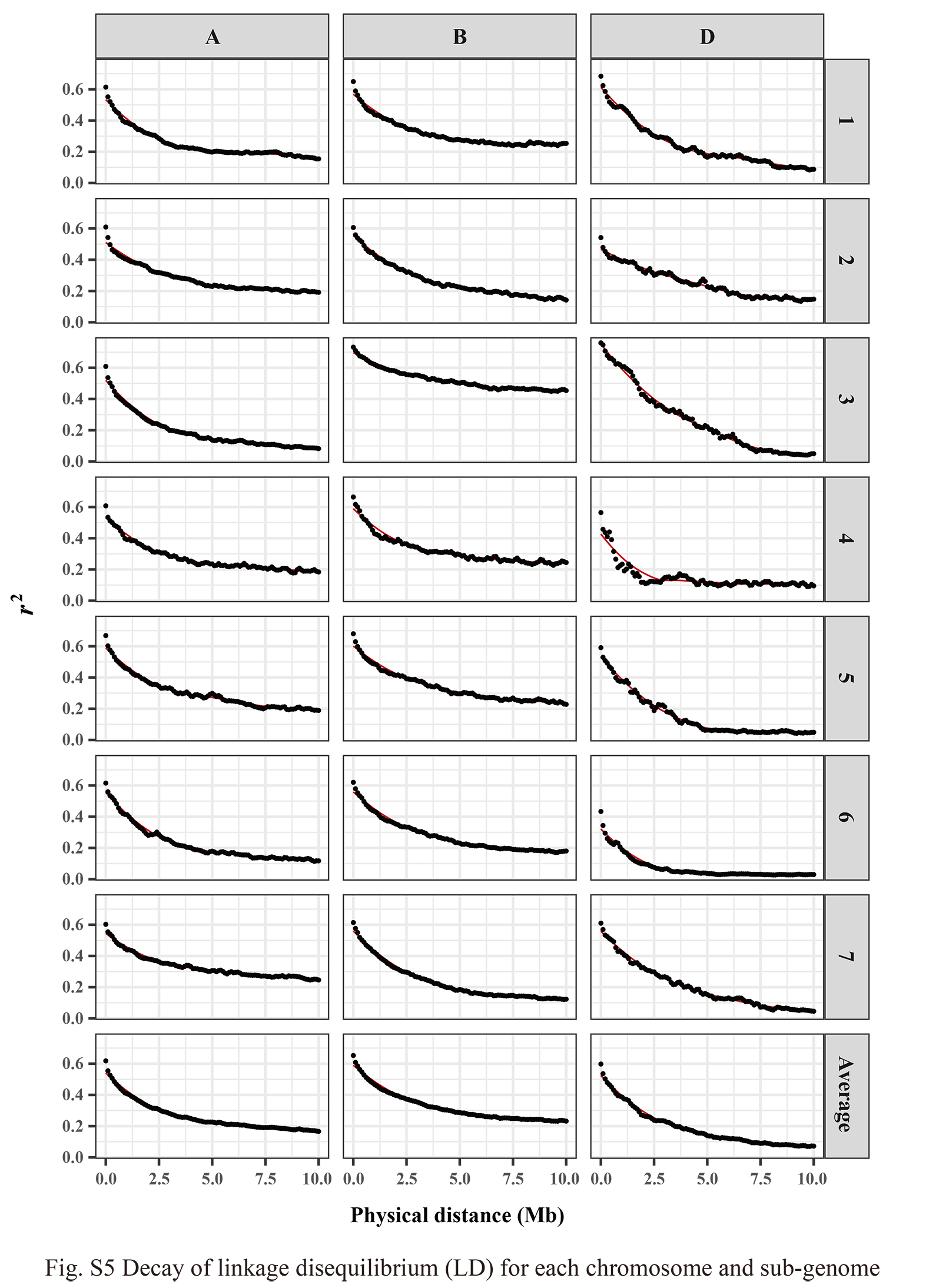

Supplement: mcz041_Suppl_Supplementary_Figure-S5 [file mcz041_suppl_supplementary_figure-s5.png]

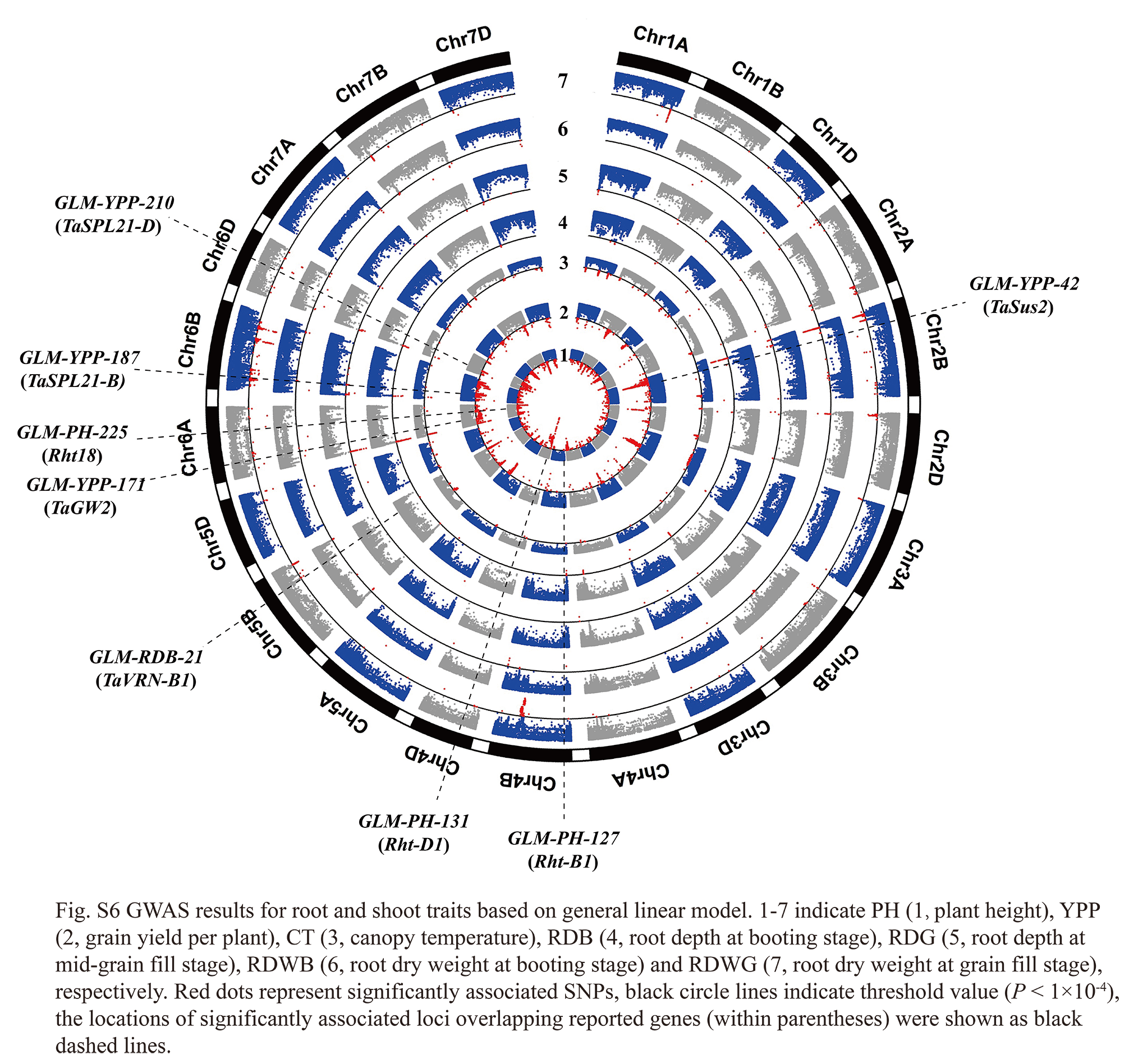

Supplement: mcz041_Suppl_Supplementary_Figure-S6 [file mcz041_suppl_supplementary_figure-s6.png]

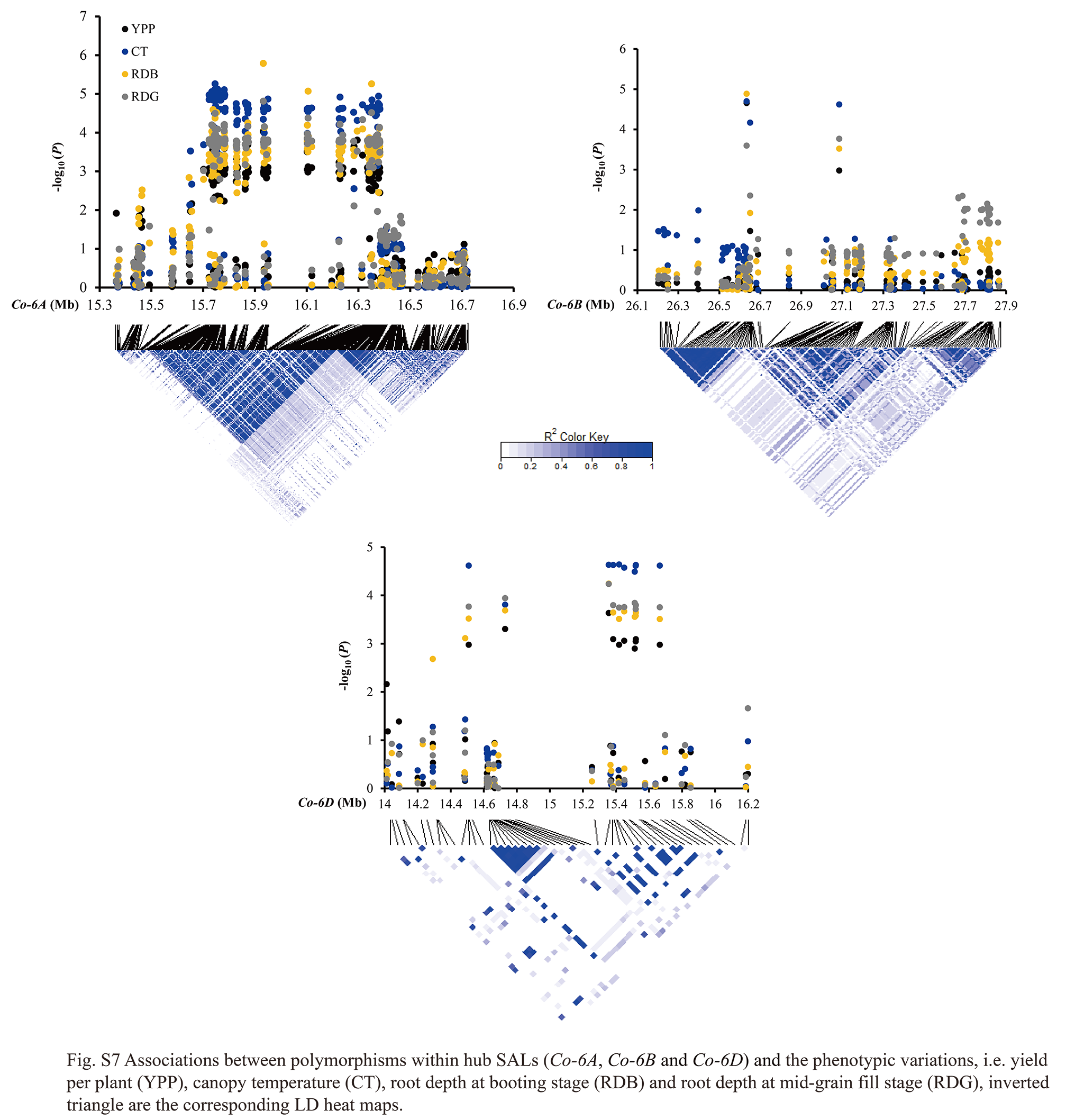

Supplement: mcz041_Suppl_Supplementary_Figure-S7 [file mcz041_suppl_supplementary_figure-s7.png]

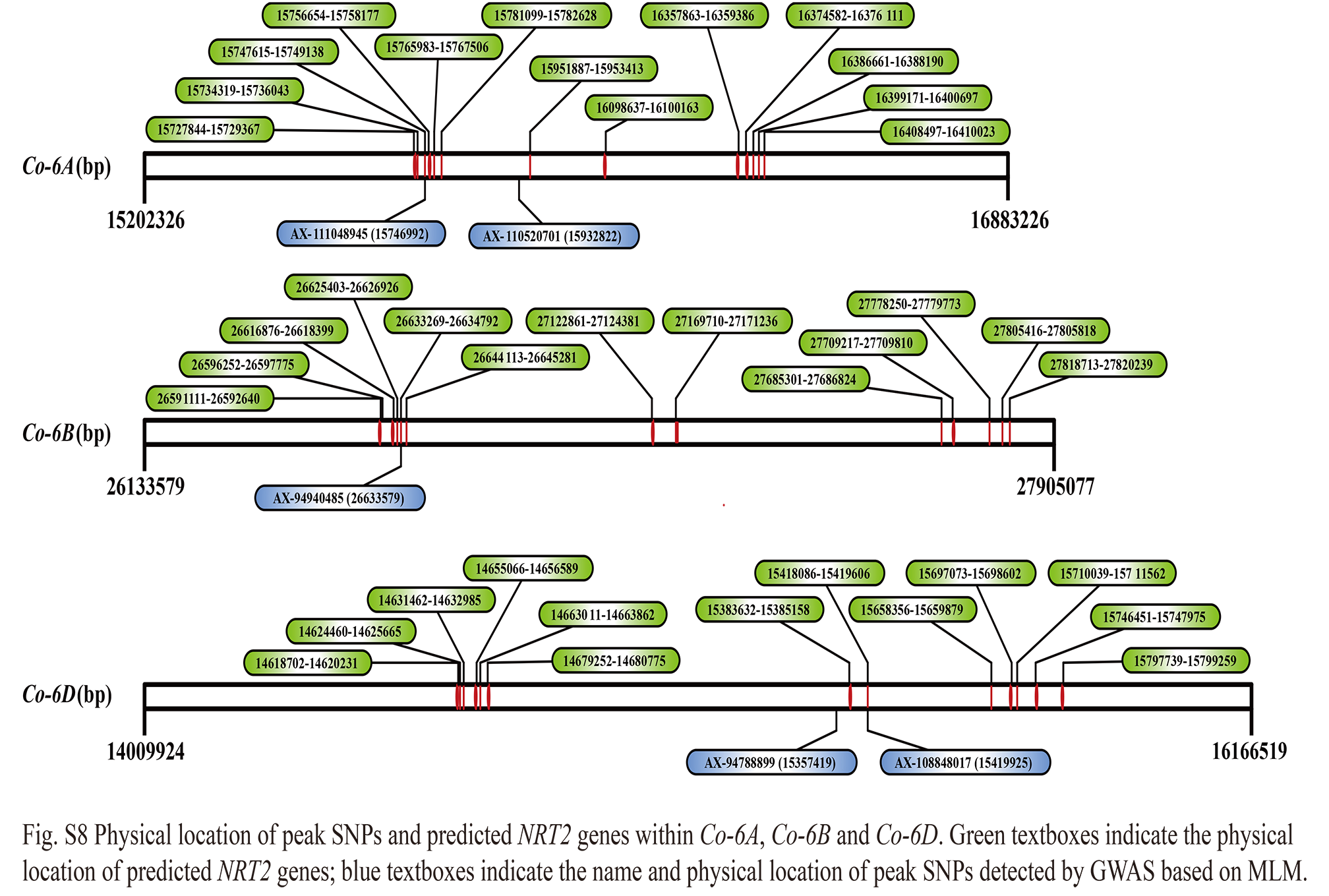

Supplement: mcz041_Suppl_Supplementary_Figure-S8 [file mcz041_suppl_supplementary_figure-s8.png]
